# Amyloid-beta-targeting monoclonal antibodies in early Alzheimer’s disease: a cochrane review summary and appraisal for the neurological community

**DOI:** 10.1007/s10072-026-09401-w

**Published:** 2026-09-17

**Authors:** Diogo Haddad Santos, Anderson Matheus Pereira da Silva

**Affiliations:** 1 Division of Neurology, MemoryHub, Cognitive and Neurodegenerative Disorders Research Group, São Paulo, SP Brazil; 2 Division of Biostatistics and Clinical Epidemiology, MemoryHub, Cognitive and Neurodegenerative Disorders Research Group, Rua Teixeira da Silva 54, Cj 71, Bela Vista, São Paulo, SP 04002-030 Brazil

**Keywords:** Alzheimer’s disease, Mild cognitive impairment, Amyloid-beta, Monoclonal antibodies, ARIA, Cochrane systematic review

## Abstract

**Background:**

Seven amyloid-beta-targeting monoclonal antibodies have been trialled in early Alzheimer’s disease. They differ in epitope and in how much plaque they clear; only two have traditional approval. A 2026 Cochrane Review pooled all seven as one class. We ask what that average tells us about any one drug.

**Methods:**

We summarise the review in mild cognitive impairment or mild dementia due to Alzheimer’s disease and report its estimates and certainty ratings unchanged. Our appraisal sits in a separate section and draws on the pivotal trials and regulatory assessments.

**Results:**

Seventeen trials (20,342 participants) were included. At 18 months the pooled standardised mean difference was −0.11 (95 % confidence interval −0.16 to −0.06) for cognition and −0.12 (−0.24 to 0.00) for dementia severity; three functional scales favoured treatment (0.09 to 0.23). Amyloid-related imaging abnormalities with oedema affected 119 versus 12 per 1,000 (risk ratio 10.02, 7.49 to 13.41). Four of the seven antibodies cleared no plaque or cleared it incompletely, so the pooled result cannot test whether clearance produces benefit. In the two approved agents dementia severity differed by 0.45 and 0.67 points, larger than the class average but below thresholds of clinical importance.

**Conclusions:**

The class average is not an estimate for any single agent. These antibodies clear amyloid and produce small differences on trial scales at 18 months, against a tenfold rise in amyloid-related oedema. Counselling should rest on agent-specific figures and a biomarker-confirmed diagnosis, and should weigh APOE ε4 genotype and antithrombotic use before the first infusion.

## Background

Alzheimer’s disease is the leading cause of dementia worldwide and a major driver of disability, dependency, and care needs in older adults [1, 2]. The clinical course begins before dementia is established and progresses through mild cognitive impairment (MCI) due to Alzheimer’s disease, followed by mild, moderate, and severe dementia. In the early symptomatic stages, patients may retain partial functional independence, which makes treatment decisions particularly relevant for patients, families, neurologists, and health systems [3, 4].

The amyloid cascade hypothesis has shaped much of the therapeutic development in Alzheimer’s disease, based on the premise that amyloid-beta accumulation initiates downstream tau pathology, neurodegeneration, and clinical decline [5, 6]. Amyloid-beta-targeting monoclonal antibodies were developed to bind different amyloid species and facilitate amyloid clearance. The clinical rationale is that reducing cerebral amyloid burden could slow cognitive and functional decline in early Alzheimer’s disease.

Recent trials of lecanemab and donanemab have renewed interest in disease-modifying treatment for Alzheimer’s disease [7, 8]. The relationship between amyloid removal and clinical benefit remains debated, however. Reported effects on cognitive and functional scales are small, while treatment requires repeated intravenous administration, biomarker confirmation, magnetic resonance imaging (MRI) surveillance, and counselling about amyloid-related imaging abnormalities (ARIA), including oedema or effusion (ARIA-E) and haemorrhagic changes (ARIA-H) [9, 10]. For neurologists, the practical question is whether these treatments offer a benefit that patients can perceive and whether the safety and monitoring burden is acceptable.

This article has two parts, and we keep them separate throughout. Part 1 reports the review as its authors wrote it, using their outcomes, their estimates, and their GRADE certainty ratings. Part 2 is our own appraisal, in which we set the class-level pooled estimate alongside agent-specific evidence, target-engagement data, and regulatory decisions. The separation is deliberate: the review has attracted substantial published criticism, and readers of a summary are entitled to know where reporting ends and interpretation begins.

## Part 1. Summary of the Cochrane Review

This article summarises the Cochrane Review *Amyloid-beta-targeting monoclonal antibodies for people with mild cognitive impairment or mild dementia due to Alzheimer’s disease*, published in the *Cochrane Database of Systematic Reviews* in 2026 [10].

### Objectives and scope

The objective was to assess the clinical benefits and harms of amyloid-beta-targeting monoclonal antibodies in people with MCI or mild dementia due to Alzheimer’s disease [10]. Nine antibodies were prespecified for inclusion: aducanumab, bapineuzumab, crenezumab, donanemab, gantenerumab, lecanemab, ponezumab, remternetug, and solanezumab.

The population comprised adults with MCI or mild dementia due to probable or possible Alzheimer’s disease, diagnosed according to recognised criteria, including the National Institute on Aging–Alzheimer’s Association recommendations and DSM criteria [3, 4]. The intervention was any amyloid-beta-targeting monoclonal antibody at any dose by parenteral route, with placebo or no treatment as the comparator. Only randomised controlled trials (RCTs) with at least 12 months of follow-up were considered, on the grounds that Alzheimer’s disease is slowly progressive and shorter trials may not capture clinical benefit or harm adequately [10].

Critical benefit outcomes were cognitive function (Alzheimer’s Disease Assessment Scale–Cognitive Subscale, ADAS-Cog), dementia severity (Clinical Dementia Rating–Sum of Boxes, CDR-SB), and functional ability (Alzheimer’s Disease Cooperative Study–Activities of Daily Living, ADCS-ADL family of instruments). Critical harm outcomes were any ARIA-E, symptomatic ARIA-E, any ARIA-H, symptomatic ARIA-H, symptomatic intracerebral haemorrhage, serious adverse events, and mortality.

### Methods and certainty assessment

The review authors searched CENTRAL, MEDLINE (PubMed), Embase, ClinicalTrials.gov, and the WHO International Clinical Trials Registry Platform, with reference and citation checking; the most recent search was 7 August 2025 [10]. Risk of bias was assessed with the Cochrane RoB 2 tool, and certainty of evidence was rated using the Grading of Recommendations, Assessment, Development and Evaluation (GRADE) approach. Where appropriate, results were synthesised using inverse-variance, random-effects meta-analysis.

### Main findings

Seventeen RCTs enrolling 20,342 participants were included; mean age ranged from 70 to 74 years. Seven studies enrolled only people with mild dementia, one enrolled only people with MCI, and the remainder enrolled mixed early Alzheimer’s disease populations. All trials were funded by the pharmaceutical industry [10]. The antibodies studied were aducanumab (three trials) [11], bapineuzumab (four) [12], crenezumab (two) [13], donanemab (one) [8], gantenerumab (four) [14], lecanemab (one) [7], and solanezumab (two) [15]. All comparators were placebo. Eleven trials lasted 18 months, four lasted 24 months, and two extended beyond 24 months [10].

Not every trial contributed to every outcome. The pooled ADAS-Cog analysis at 18 months drew on 9,895 participants from 13 trials and the CDR-SB analysis on 8,053 participants from 9 trials, so the two headline cognitive outcomes rested on 49% and 40% of the randomised total respectively, on our calculation from the participant counts reported in the review [10].

### Effects on cognition, dementia severity, and function

At 18 months, amyloid-beta-targeting monoclonal antibodies probably result in little or no difference in cognitive function measured by ADAS-Cog. The pooled standardised mean difference (SMD) was − 0.11 (95% CI − 0.16 to − 0.06; 13 studies; 9,895 participants; moderate-certainty evidence) [10]. The review authors back-converted this to approximately 0.85 points on ADAS-Cog and judged that magnitude trivial [10, 16]. The conversion is not a direct measurement: it is the SMD multiplied by a reference standard deviation, and the resulting figure moves with the standard deviation chosen. Readers who prefer to reason in the original units may consult the trial-level differences for the two currently approved agents, given in Table 1.

**Table 1:** Class-level pooled estimates from the Cochrane Review compared with agent-specific estimates in original units from the two pivotal trials of currently approved antibodies. Class-level values are standardised mean differences across seven antibodies, except for ARIA-E, which is a risk ratio; trial-level values are adjusted mean differences on the scale named, except for ARIA-E, which is the incidence among treated participants. The two columns answer different questions and should not be read as alternative estimates of the same quantity

Outcome	Class-level Cochrane estimate (95% CI) [10]	Lecanemab, CLARITY-AD, 18 months (95% CI) [7]	Donanemab, TRAILBLAZER-ALZ 2, 76 weeks (95% CI) [8]
Registered primary endpoint	Not applicable	CDR-SB	iADRS
Cognition	ADAS-Cog − 0.11 (− 0.16 to − 0.06); 13 studies, 9,895 participants	ADAS-Cog14 − 1.44 (− 2.27 to − 0.61)	ADAS-Cog13 − 1.52 (− 2.25 to − 0.79), low/medium tau
Dementia severity	CDR-SB − 0.12 (− 0.24 to 0.00); 9 studies, 8,053 participants	CDR-SB − 0.45 (− 0.67 to − 0.23)	CDR-SB − 0.67 (− 0.95 to − 0.40), low/medium tau
Composite	Not analysed	ADCOMS − 0.050 (− 0.074 to − 0.027)	iADRS 3.25 (1.88 to 4.62), low/medium tau
Function	ADCS-ADL 0.09 (0.03 to 0.16); ADCS-ADL-MCI 0.23 (0.12 to 0.33)	ADCS-ADL-MCI 2.0 (1.2 to 2.8)	Not reported in comparable units
Any ARIA-E	RR 10.02 (7.49 to 13.41); 11 studies, 13,595 participants	12.6% of treated participants	24.0% of treated participants

For dementia severity measured by CDR-SB, amyloid-beta-targeting monoclonal antibodies may result in little or no difference compared with placebo (SMD − 0.12, 95% CI − 0.24 to 0.00; 9 studies; 8,053 participants; low-certainty evidence), corresponding on the same back-conversion to about 0.29 points on CDR-SB [10].

Three functional instruments were analysed separately, and all three favoured treatment with confidence intervals excluding the null: ADCS-ADL (SMD 0.09, 95% CI 0.03 to 0.16; 3 studies; 3,478 participants; moderate-certainty evidence), ADCS-iADL (SMD 0.21, 95% CI 0.10 to 0.32; 1 study; 1,252 participants; low-certainty evidence), and ADCS-ADL-MCI (SMD 0.23, 95% CI 0.12 to 0.33; 4 studies; 2,802 participants; low-certainty evidence) [10]. The direction is therefore consistent across instruments; what remains uncertain is the size of the difference and whether it is large enough to matter to patients. The review authors described the effect on functional ability as small at best [10].

A note on terminology is warranted for readers who do not work in evidence synthesis. In GRADE and Cochrane usage, “little or no difference” is a statement about the magnitude of an estimated effect relative to a threshold of importance, not a statement that no effect was observed. The ADAS-Cog and ADCS-ADL intervals above exclude the null; the phrase conveys the review authors’ judgement that the effect is too small to matter, which is a separate question from whether it is distinguishable from zero.

### Safety profile: ARIA and serious adverse events

The safety findings were most consistent for ARIA-E. Amyloid-beta-targeting monoclonal antibodies increased the occurrence of any ARIA-E: 119 per 1,000 people treated versus 12 per 1,000 receiving placebo (risk ratio, RR 10.02, 95% CI 7.49 to 13.41; 11 studies; 13,595 participants; moderate-certainty evidence) [10]. Symptomatic ARIA-E was also more frequent with treatment: 30 per 1,000 people treated versus 1 per 1,000 receiving placebo (RR 52.49, 95% CI 40.13 to 68.66; 2 studies; 3,522 participants; moderate-certainty evidence) [10].

Three trials reported any ARIA-H at 18 months, but heterogeneity precluded pooling; individual risk ratios were 2.31 (95% CI 1.90 to 2.80), 1.91 (95% CI 1.49 to 2.46), and 0.85 (95% CI 0.47 to 1.52) [10]. Symptomatic ARIA-H was reported in one trial (RR 3.00, 95% CI 0.61 to 14.81; 1 study; 1,795 participants), which the review rated as moderate-certainty evidence [10]. At 18 months, serious adverse events were similar between groups (RR 1.04, 95% CI 0.94 to 1.16; 9 studies; 11,904 participants; high-certainty evidence), and the review reported no evidence of a difference in mortality (RR 1.17, 95% CI 0.74 to 1.86; 7 studies; 9,733 participants; high-certainty evidence) [10]. We comment on these certainty ratings in Part 2.

A methodological issue highlighted by the review authors was functional unblinding. Although the trials were nominally double-blind, adverse events such as infusion reactions or ARIA may allow participants, clinicians, or assessors to infer treatment allocation [10, 17, 18].

## Review authors’ conclusions

The review authors concluded that the effect of amyloid-beta-targeting monoclonal antibodies on cognitive function and dementia severity at 18 months is trivial in people with MCI or mild dementia due to Alzheimer’s disease, that the effect on functional ability is small at best, and that these drugs increase the risk of ARIA. They further concluded that successful amyloid clearance from the brain does not appear to translate into clinically meaningful effects in this population, and that future research on disease-modifying therapies should explore mechanisms beyond amyloid removal [10]. This last inference is the most contested element of the review, and we address it below.

## Part 2. Appraisal of the Cochrane Review

### Scope of this appraisal

Everything that follows is our own appraisal and is not content of the Cochrane Review. Neither author took part in the review, and there is no overlap of authorship, institution, or funding between this article and the review team; our competing-interest statement is unchanged. We have tried to be even-handed: the review is, as far as we can judge, a careful and correctly executed synthesis of the question it set itself, and not all of the objections raised against it since publication are of equal weight. The objection we do consider decisive is narrower, and concerns one inferential step rather than the analyses.

### How much amyloid was actually removed

The review’s mechanistic conclusion concerns the relationship between amyloid clearance and clinical change, but its analyses measure only the clinical side of that relationship. The biomarker side varies widely across the class, and setting out what it shows is a precondition for evaluating the relationship at all. Table 2 summarises target engagement for the seven antibodies contributing data.

**Table 2:** Target engagement of the seven antibodies contributing data to the Cochrane Review. Values are taken from the pivotal trial reports and from regulatory product information; the review did not analyse amyloid PET as an outcome. Centiloid values are unavailable for bapineuzumab and crenezumab; amyloid-negativity thresholds are expressed in SUVR for aducanumab and in centiloids for the remaining agents

Antibody	Trials in the review	Amyloid PET result	Reaching amyloid negativity
Donanemab	1	−88.0 centiloids from baseline at 76 weeks (95% CI − 90.20 to − 85.87), low/medium tau, versus + 0.2 under placebo [8]	80.1% below 24.1 centiloids [8]
Lecanemab	1	−59.1 centiloids versus placebo at 18 months (95% CI − 62.6 to − 55.6); treated group mean 23.0 centiloids [7]	Not reported in the pivotal publication; the product information states 67% below 30 centiloids after week 79 [41]
Aducanumab (high dose)	3	−64.2 centiloids (EMERGE) and − 53.5 centiloids (ENGAGE) versus placebo at 78 weeks [11, 19]	48% and 31% (SUVR ≤ 1.10) [11]
Gantenerumab	4	−66.44 (95% CI − 74.71 to − 58.16) and − 56.46 centiloids (95% CI − 64.36 to − 48.56) versus placebo at 116 weeks [14]	28.0% and 26.8% (< 24 centiloids) [14]
Bapineuzumab	4	No reduction; +0.001 SUVR in treated APOE ε4 carriers versus + 0.102 under placebo, difference − 0.101 (*P* = 0.004) [12]	Not applicable
Solanezumab	2	No reduction in fibrillar plaque [15]; in a separate trial in preclinical disease, not included in the review, amyloid rose in both arms, with a difference in accumulation of 7.7 centiloids (95% CI 5.1 to 10.4) [20]	Not applicable
Crenezumab	2	No significant change on florbetapir PET [13]	Not reported

At the high-clearance end, donanemab reduced amyloid burden by 88.0 centiloids from baseline at 76 weeks in the low/medium-tau population (95% CI 85.87 to 90.20), against an increase of 0.2 centiloids under placebo, and 80.1% of treated participants fell below the 24.1-centiloid amyloid-negativity threshold [8]. Lecanemab reduced amyloid by 59.1 centiloids relative to placebo at 18 months (95% CI 55.6 to 62.6), bringing the treated group to a mean of 23.0 centiloids, below the positivity threshold used in that trial [7]. High-dose aducanumab reduced amyloid by 64.2 and 53.5 centiloids relative to placebo at 78 weeks in EMERGE and ENGAGE respectively [11, 19].

The remaining agents behaved differently. Gantenerumab lowered amyloid relative to placebo at 116 weeks by 66.44 centiloids (95% CI 58.16 to 74.71) in GRADUATE I and by 56.46 centiloids (95% CI 48.56 to 64.36) in GRADUATE II, yet only just over a quarter of treated participants reached amyloid negativity, and the trial investigators described the plaque removal as smaller than expected [14]. Bapineuzumab produced no clearance: among APOE ε4 carriers the treated group was essentially flat on Pittsburgh compound B positron emission tomography (PET), with a change of + 0.001 in standardised uptake value ratio (SUVR), while the placebo group accumulated (+ 0.102), a difference of − 0.101 (*P* = 0.004) that represents arrest of accumulation rather than removal [12]. Solanezumab, which binds monomeric amyloid-beta, did not reduce fibrillar plaque in EXPEDITION3 [15]; in the A4 trial in preclinical disease, which the review did not include, amyloid rose in both arms, with a between-group difference in accumulation of 7.7 centiloids (95% CI 5.1 to 10.4) [20]. Crenezumab produced no significant change on florbetapir PET [13].

Four of the seven antibodies contributing data therefore either failed to remove plaque or removed it incompletely. The distinction between high-clearance and low-clearance immunotherapies is not new and has been used to define the class in earlier syntheses [21]. This does not invalidate any pooled estimate. It does determine what that estimate can be used to argue.

### Class-level pooling and the inference about clearance

Two claims in this literature cannot both be maintained. The first is that successful amyloid clearance does not translate into clinically meaningful effects. The second is that pooling agents of very different potency yields a class average less informative than agent-specific estimates. If the class average is diluted by antibodies that never cleared plaque, that average cannot serve as a test of what follows when plaque is cleared.

Of the two, we hold the second. The review’s clinical estimates answer the question the review prespecified, which is the average effect of a therapeutic class. The conclusion drawn from them about amyloid clearance is a claim of a different kind, and the analyses presented do not support it, because the antibodies that produced the clearance are a minority within the pooled set and were not analysed separately. We therefore report the review authors’ conclusion because it is theirs, and we do not endorse the mechanistic step. Similar objections have been raised in several published commentaries, including one in this journal [22–24].

A caveat is owed on the instrumental-variable evidence. An analysis published in 2021 [25] has been cited in support of the claim that benefit is limited, including for higher-clearance agents. That citation is inappropriate: the analysis rested on data available to its authors in April 2020 and could not have included CLARITY-AD or TRAILBLAZER-ALZ 2. The same group has since published a Bayesian update incorporating instrumental-variable estimates derived from CLARITY-AD, TRAILBLAZER-ALZ 2, and GRADUATE I and II, and concluded that across a wide range of prior beliefs a reader should infer a small cognitive benefit of amyloid reduction within the trial time frame, with a posterior estimate of roughly − 0.06 CDR-SB points per 10-centiloid reduction and with large benefits implausible [26]. An independent instrumental-variable analysis on corrected data, published before that update by authors affiliated with one manufacturer, reported a reduction in CDR-SB decline of 0.09 points per 0.1-unit decrease in SUVR (95% CI 0.034 to 0.15) [27]. These analyses rest on strong assumptions, in particular that the drug affects cognition only through amyloid, and their proponents have themselves catalogued the limitations of aggregate-data instrumental-variable estimation [28].

### Reconciling the class-level estimate with regulatory decisions

Neurologists will reasonably ask how a Cochrane Review can report little or no difference when regulators granted traditional approval to lecanemab and donanemab on the strength of trials included in that same review. Four differences account for most of the gap.

The first is the unit of assessment. Regulators evaluated one dossier per agent; the review evaluated a class of nine prespecified antibodies. Seven contributed data, of which four have never been approved in any jurisdiction and a fifth, aducanumab, was withdrawn from the market in 2024. Restricting synthesis to agents that had been approved increases the magnitude of the estimate, though less than is sometimes implied. A meta-analysis limited to lecanemab and aducanumab reported a pooled CDR-SB SMD of − 0.14 (95% CI − 0.24 to − 0.03) and an ADAS-Cog SMD of − 0.14 (95% CI − 0.20 to − 0.08) [29]. The corresponding class-level values in the review are − 0.12 (95% CI − 0.24 to − 0.00) and − 0.11 (95% CI − 0.16 to − 0.06) [10], so restriction increases the magnitude of the estimates by roughly one sixth and a little over one quarter, with intervals that overlap substantially. Two caveats limit what the comparison shows. That analysis predates the approval of donanemab and includes aducanumab, so it is not a synthesis of the agents now in use; and its harm estimates are reported as odds ratios, which for an outcome as common as ARIA-E are not directly comparable with the review’s risk ratios. The defensible conclusion is narrower than the arithmetic suggests: restriction is not a device for flattering these drugs.

The second is endpoint choice. ADAS-Cog, the review’s headline cognitive outcome, was the registered primary endpoint of neither pivotal trial. CLARITY-AD was powered on CDR-SB and reported an adjusted mean difference of − 0.45 (95% CI − 0.67 to − 0.23) at 18 months [7]. TRAILBLAZER-ALZ 2 was powered on the integrated Alzheimer’s Disease Rating Scale (iADRS) and reported a difference of 3.25 (95% CI 1.88 to 4.62) in the low/medium-tau population, with a CDR-SB difference of − 0.67 (95% CI − 0.95 to − 0.40) [8]. A synthesis organised around ADAS-Cog is answering a question that neither regulatory submission posed.

The third is the standard of judgement. GRADE evaluates one outcome at a time against a threshold of importance and downgrades for imprecision, inconsistency, and risk of bias. A regulatory assessment weighs an entire dossier, including concordance across endpoints, dose–response, and target engagement, against an unmet need and a specified risk-management plan. The two procedures can reach different conclusions from identical data without either being mistaken.

The fourth is that the regulatory picture is not uniform, and describing it simply as approval understates the divergence. The European Medicines Agency issued an initial negative opinion for both agents and authorised them only after re-examination, with indications restricted to APOE ε4 non-carriers and heterozygotes [30, 31]. In England, the National Institute for Health and Care Excellence issued final draft guidance in June 2025 recommending neither drug for routine use; appeals against both recommendations were heard and the appraisals reopened in March 2026, and final guidance had not been published when this article was prepared [32]. The disagreement is therefore not between Cochrane and regulators as monolithic blocs, but among assessors applying different thresholds to the same trials.

### Effect measures, units, and minimal clinically important differences

Combining ADAS-Cog and CDR-SB as standardised mean differences is defensible when the aim is a single summary across instruments, but it carries a cost. Both are familiar scales normally reported in their own units, and standardisation removes the anchor that allows a clinician to judge whether a difference matters. Back-conversion restores the units only in appearance, since the product depends on the reference standard deviation adopted, and that parameter is not itself a trial result. The dependence is easy to quantify. An SMD of − 0.11 rendered as 0.85 ADAS-Cog points implies a reference standard deviation of about 7.7 points; the same SMD would yield 0.66 points against a standard deviation of 6 and 0.99 points against one of 9. Applying that implied standard deviation to the confidence limits places the class-level effect between about 0.5 and 1.2 ADAS-Cog points. The CDR-SB back-conversion implies, on the same reasoning, a reference standard deviation of about 2.4 points. Whether 0.85 points is called trivial therefore rests in part on a modelling choice the reader cannot see.

The threshold against which any of these values is judged is itself stage-dependent. Minimal clinically important differences (MCIDs) for CDR-SB have been estimated at approximately 1 point in MCI due to Alzheimer’s disease and 2 points in mild dementia [33], with corresponding ADAS-Cog estimates of 2 to 3 points in MCI and 3 points in mild dementia [16]; the review itself applied a 4-point threshold at the dementia stage [10]. One included trial enrolled participants with MCI only and seven enrolled participants with mild dementia only, so the pooled estimate spans populations whose yardstick differs by a factor of two. Stage-stratified pooled estimates were not reported, and without them the class estimate cannot be compared against any single threshold.

Consistency about these thresholds is also required. It is not coherent to call an effect trivial by reference to an MCID and, in the same argument, to hold that MCID thresholds derived from symptomatic drugs may not capture the value of disease modification. Our position is that current MCID estimates are the best available yardstick and should be used, while acknowledging that they were derived for change on symptomatic treatment and may not capture the value of a therapy intended to alter a trajectory. On that yardstick, the class-level estimates and the agent-specific estimates for lecanemab and donanemab all fall below conventional thresholds at 18 months. We accordingly describe these effects as small and of uncertain clinical importance, and we avoid the words trivial and absent, which import a judgement the data do not settle.

### Certainty, precision, and the symmetry of scepticism

Two of the review’s certainty ratings deserve comment. The mortality result is rated high-certainty evidence, yet an RR of 1.17 with an interval from 0.74 to 1.86 is compatible with anything from a 26% reduction to an 86% increase in deaths. That is an absence of evidence of a difference rather than evidence of no difference, and the analysis is underpowered for an outcome as infrequent as death over 18 months. Deaths occurring in the context of ARIA have been reported in the donanemab pivotal trial and in the lecanemab open-label extension [8, 34], which makes precision on this outcome clinically consequential.

Symptomatic ARIA-H rests on a single trial with an RR of 3.00 and an interval from 0.61 to 14.81, spanning more than a twenty-fold range and crossing the null. GRADE advises considering a two-level downgrade for imprecision when events are very few and the interval spans both appreciable benefit and appreciable harm, for which relative risks of 0.75 and 1.25 are offered as a rough guide [35]. Both conditions are met here, and from the starting point of high certainty for randomised evidence that reasoning gives low certainty. A moderate rating is difficult to reconcile with it, and we would read this outcome as low certainty.

The heterogeneity that prevented pooling of the any-ARIA-H outcome, with trial-level risk ratios of 2.31, 1.91, and 0.85, plausibly reflects the same between-agent differences that we invoke against class-level benefit estimates. Consistency requires acknowledging that antibodies too different to average for benefit are also too different to average for harm. The practical consequence is that ARIA risk should be quoted per agent rather than as a class figure.

The same requirement of symmetry applies to functional unblinding, which is often invoked one-sidedly. The direction and magnitude of any such bias are unknown, and unblinding may inflate or attenuate an observed difference. It bears on any outcome ascertained with a subjective component, which includes the clinical rating scales and the classification of an imaging finding as symptomatic; it bears less on the radiological detection of ARIA, which follows protocol-scheduled MRI with central reading. Unblinding is therefore a general limitation of this evidence base rather than an argument directed at the benefit estimates alone. The same reasoning applies to industry sponsorship, which is a feature of all 17 included trials and cannot selectively threaten one side of the ledger.

### What the review does not resolve

Three limitations belong to the review as it stands rather than to a future research agenda. The first is analytic coverage. Fewer than half of the randomised participants informed the headline cognitive analyses, 9,895 of 20,342 for ADAS-Cog and 8,053 of 20,342 for CDR-SB. This reflects which trials administered which instruments, but a gap of that size bears on reporting bias and on the certainty ratings that depend on it.

The second is the absence of stratified estimates. The clinically decisive question is not whether an average patient benefits but whether identifiable subgroups do, defined by disease stage, baseline tau burden, and APOE ε4 status. APOE ε4 carriage modifies ARIA risk steeply and with a gene-dose effect [36], and low baseline tau has been associated with larger treatment differences in post-hoc analyses [8]. Pooled estimates stratified by these variables were not reported. An average that conceals responders is not evidence that none exist, and this is a present limitation of the review rather than a topic for future work.

The third is the treatment of long-term data. Extension data now exist and bear on whether the early separation grows. In the CLARITY-AD open-label extension, time to worsening on CDR-SB through 36 months favoured early over delayed start (hazard ratio 0.70, 95% CI 0.59 to 0.84), and the delayed-start group did not catch up [34]. In the long-term extension of TRAILBLAZER-ALZ 2, which retained blinding, the early-start group differed from a propensity-weighted external control by − 1.2 CDR-SB points at 36 months (95% CI − 1.7 to − 0.7), while the within-trial comparison of early against delayed start gave a hazard ratio of 0.73 (95% CI 0.62 to 0.86) for progression to the next clinical stage [37].

These findings are the sponsors’ own analyses and should be read with care, and the two programmes differ in ways that matter. The CLARITY-AD extension was open-label; the TRAILBLAZER-ALZ 2 extension retained blinding of participants and investigators, which makes its within-trial contrast the more secure of the two. In both, the early versus delayed comparison preserves the original randomisation, whereas the comparisons against externally derived cohorts do not. Neither programme used a formal delayed-start design with prespecified non-inferiority testing; the analyses were exploratory and not adjusted for multiplicity; and long-term follow-up was incomplete, with 464 of the 1,616 participants who received lecanemab having reached 36 months of exposure at the reported data cut [34]. A modelling study calibrated to the published summary statistics reported that decline in the early-start groups was steeper during extension follow-up than during the double-blind period, approximating the double-blind placebo slopes [38]; it rests on aggregate rather than individual data, and one of its authors is employed by a pharmaceutical company. The defensible summary is that the gap against externally derived controls widens over time while the rate of decline within the treated groups does not slow, and that extensions of this design cannot distinguish a continuing treatment effect from an unrepresentative external comparator. That is a more useful message for neurologists than a presentation of the 18-month horizon as unexamined.

### Implications for clinical neurology

For neurologists, the review provides a framework for counselling, provided the class-level estimate is not read as an estimate for the agent they are considering. The class-level synthesis indicates that, averaged across seven antibodies of widely differing potency, the difference from placebo on cognitive and dementia-severity scales at 18 months is small while the increase in ARIA-E is large. For a patient being considered for lecanemab or donanemab, the relevant figures are the agent-specific ones in Table 1, which are larger for benefit and larger for harm alike.

What the two readings share is that the expected difference over 18 months is smaller than what patients and families usually have in mind when they ask whether treatment will preserve memory, independence, daily activities, or time at home. That conversation changes little between the class estimate and the agent estimate. What changes substantially is the risk figure, which should be quoted per agent.

The elements of a treatment pathway set out below do not derive from the Cochrane Review, which compared antibodies with placebo and did not evaluate service delivery. They come from appropriate-use recommendations, regulatory product information, and consensus guidance, and we cite them as such. Biological confirmation of Alzheimer’s disease is required before treatment [39, 40]. APOE ε4 genotyping is recommended before initiation to inform ARIA risk, and in the European Union the authorised indications for both agents exclude ε4 homozygotes [30, 31, 39, 40]. Baseline MRI and scheduled surveillance MRI are specified in both product labels, on schedules that differ between agents [41, 42], and standardised acquisition and reporting protocols have been recommended [43]. Baseline vascular burden matters: white-matter hyperintensity severity and superficial siderosis are associated with ARIA-E risk, with a weaker association for microhaemorrhage count [44]; more than four microhaemorrhages, cortical superficial siderosis, or a major vascular contribution to cognitive impairment is a ground for exclusion under current appropriate-use recommendations [40]. Anticoagulation is a specific concern: intracerebral haemorrhage occurred in 2.5% of lecanemab-treated patients who were receiving an anticoagulant and in none of the anticoagulant-treated patients assigned to placebo [41], and the European product information states that treatment should not be initiated in patients receiving ongoing anticoagulant therapy [30]. Dedicated vascular neurology guidance addresses antithrombotic use, thrombolysis, and the management of severe ARIA in treated patients [45]. In Italy, an intersocietal document convened by the Italian Society of Neurology sets out the requirements for prescribing and infusion centres [46].

These requirements are not evenly attainable. Where amyloid PET, cerebrospinal fluid or validated blood biomarkers, serial MRI at adequate field strength, infusion capacity, and specialist follow-up are limited, the binding constraint is delivery rather than efficacy. In a European survey of neurologists and neuroradiologists, 82% of respondents reported that their centre had no dedicated ARIA imaging protocol [47]. In Brazil, the Brazilian Academy of Neurology has published a position paper on candidate selection and on barriers to diagnosis [48], and the obstacles to delivery within the public health system have been described separately [49]. Blood-based biomarkers may ease the diagnostic bottleneck but do not address monitoring capacity [50].

The review does not establish that amyloid-beta-targeting monoclonal antibodies lack biological activity, and the target-engagement data summarised in Table 2 exclude that reading for the high-clearance agents. What it establishes is that the class average on clinical scales at 18 months is small. Whether the agent-specific difference is worth the monitoring burden and the ARIA risk for a particular patient depends on stage, genotype, vascular status, concomitant medication, frailty, access, and what that patient is hoping to gain. Shared decision-making based on absolute effects, residual uncertainty, harms, and patient priorities remains the appropriate frame.

### Implications for future research

Future evidence synthesis would benefit from looking beyond a single class-level estimate. Agent-specific analyses, or prespecified subgroups stratified by clearance achieved, disease stage, tau burden, and APOE ε4 status, would be more informative for current practice than an undifferentiated pooled estimate [29, 51]. Where a therapeutic class contains members that failed to engage the target, reporting the target-engagement data alongside the clinical data allows readers to judge the mechanistic question for themselves. Reporting should remain transparent on symptomatic and asymptomatic ARIA, and thresholds for clinically meaningful change should be prespecified and stage-specific [16, 33, 52]. Greater attention to external validity is also needed, including representation of populations under-represented by race and ethnicity, educational and socioeconomic diversity, vascular comorbidity, frailty, and feasibility across health systems.

Follow-up beyond 18 months requires designs that can bear the weight of the question. The available extension data are informative, but their limitations differ according to the comparison being made. The early-versus-delayed contrasts retain the original randomisation, and are weakened by exploratory analysis, absence of adjustment for multiplicity, and incomplete long-term follow-up rather than by the absence of a control group; the comparisons against externally derived cohorts are uncontrolled, and further open-label follow-up of that kind will not settle whether early separation accumulates. Randomised withdrawal, formal delayed-start designs with prespecified non-inferiority testing, and registry-based effectiveness cohorts with contemporaneous comparators would be more decisive. Two randomised trials in preclinical Alzheimer’s disease, TRAILBLAZER-ALZ 3 and AHEAD 3–45, are still in follow-up and have not reported; those trials, rather than post-hoc subgroups, are the proper test of whether earlier treatment yields larger benefit.

Research should also examine non-amyloid mechanisms, combination approaches, and interventions targeting the broader biology of Alzheimer’s disease, including tau pathology, neuroinflammation, synaptic dysfunction, vascular injury, and cognitive resilience. The relevant questions are shifting from whether amyloid can be removed to which patients derive perceptible benefit from its removal, when treatment should begin, whether maintenance after clearance is warranted, and whether combined targets add benefit.

### Conclusion

The Cochrane Review offers a careful class-level synthesis showing that, averaged across seven amyloid-beta-targeting antibodies, the difference from placebo on cognitive, dementia-severity, and functional scales at 18 months is small, while ARIA-E is markedly more frequent. Neurologists should not read that average as an estimate for lecanemab or donanemab specifically, and should not read the review’s conclusion about amyloid clearance as having been tested by the analyses presented, since most of the antibodies in the pooled set either did not clear plaque or cleared it incompletely. The agent-specific estimates are larger for benefit and larger for harm; for both agents they remain below conventional thresholds of clinical importance at 18 months, and both carry a monitoring burden that many health systems are not yet equipped to provide. That is a narrower conclusion than either the review’s headline or its critics’ rejoinders, and a more useful one at the bedside.

## Data Availability

Data sharing is not applicable to this article, as no new datasets were generated or analysed. The underlying Cochrane Review (CD016297) and its data are publicly available through the *Cochrane Database of Systematic Reviews* at https://doi.org/10.1002/14651858.CD016297.

## Declarations

### Use of large language models or other AI tools

No generative artificial-intelligence tool was used for content creation, analysis, or interpretation.
